# Characteristics of Older Adults with Favorable Locomotive Syndrome and Frailty Outcomes Following Locomotion Training: A Preliminary Pre–Post Study

**DOI:** 10.3390/jcm15197748

**Published:** 2026-10-08

**Authors:** Ikki Yoshida, Yohei Sawaya, Tamaki Hirose, Yuta Hanawa, Hiroto Ito, Reina Ohkubo, Ryo Sato, Tomohiko Urano

**Affiliations:** 1 Department of Rehabilitation, International University of Health and Welfare SHIOYA Hospital, 77 Tomita, Yaita 329-2145, Tochigi, Japan; 2 Division of Physical Therapy, Doctoral Program in Health Sciences, Graduate School of Health and Welfare Sciences, International University of Health and Welfare, 2600-1 Kitakanemaru, Otawara 324-8501, Tochigi, Japan; 3 Department of Physical Therapy, School of Health Sciences, International University of Health and Welfare, 2600-1 Kitakanemaru, Otawara 324-8501, Tochigi, Japan; 4 Department of Rehabilitation, International University of Health and Welfare Nasu Medical Center, 537-3 Iguchi, Nasushiobara 329-2763, Tochigi, Japan; 5 Motegi Town Hall, Health and Welfare Division, 155 Motegi, Motegi, Haga 321-3598, Tochigi, Japan; 6 Department of Geriatric Medicine, School of Medicine, International University of Health and Welfare, 4-3 Kozunomori, Narita 286-8686, Chiba, Japan

**Keywords:** elderly, locomotive syndrome, locomotion training

## Abstract

**Background/Objectives:** We evaluated changes in locomotive syndrome (LS) and frailty following locomotion training (LT) in community-dwelling older adults and explored their characteristics with favorable outcomes for each condition. **Methods:** In this pre–post intervention study, 37 community-dwelling older adults (mean age, 77.6 ± 6.0 years) who participated in a community-based preventive care program completed a 126-day home-based LT program of single-leg standing, squats, and heel raises. Before and after the intervention, LS was assessed using the LS risk test and frailty with the Japanese version of the Cardiovascular Health Study (J-CHS) criteria and Questionnaire for Medical Checkup of Old-Old (QMCOO); changes in LS and frailty were analyzed, and the baseline characteristics of participants with favorable outcomes were explored. **Results:** The LT was performed as prescribed or partially on 84.8% of days. Following the intervention, the LS stage (*p* = 0.016), J-CHS score (*p* = 0.014), and usual walking speed (*p* < 0.001) significantly improved. The QMCOO total score did not change significantly. Individual-level LS stage improved by at least one stage in 20.6% of participants with baseline LS. In participants with baseline pre-frailty/frailty, 43.5% (J-CHS) and 31.3% (QMCOO) improved by at least one category. LS favorable status was not associated with baseline frailty measures. In both J-CHS and QMCOO assessments, the robust maintenance/improvement groups were younger and had better baseline two-step test values and GLFS-25 scores. The QMCOO robust maintenance/improvement group had a better LS stage. **Conclusions:** Following LT, LS stage and J-CHS total score improved significantly, with favorable individual-level outcomes in LS and frailty. Participants with favorable frailty outcomes had better baseline LS measures, whereas participants with favorable LS outcomes did not differ in baseline frailty measures. Large controlled studies are warranted.

## 1. Introduction

In Japan, population aging constitutes a major social issue owing to the growing number of older adults who require long-term care [1]. Early identification of functional decline and timely intervention are essential for maintaining independence among older adults. In this context, frailty and the locomotive syndrome (LS) have received increasing attention. Frailty is defined as a state of increased vulnerability to stressors resulting from an age-related decline in physiological reserve [2]. It is a multidimensional condition that encompasses the physical, psychological, cognitive, and social domains [2]. In contrast, LS is defined as a condition that is characterized by impaired mobility owing to disorders of the locomotive organs and specifically focuses on musculoskeletal dysfunction [3]. Although frailty and LS are distinct concepts with different diagnostic criteria, they are considered potentially reversible with appropriate intervention [4]. Furthermore, LS has been proposed to develop before and contribute to the progression of physical frailty [4]. A longitudinal study reported that older adults with LS stage 2 or 3 were more likely to experience progression of physical frailty over 1 year than those without LS [5]. Therefore, early intervention for LS may help prevent the progression of physical frailty, which underscores the importance of interventions targeting LS to maintain the health and functional independence of older adults.

To prevent and improve LS, the Japanese Orthopaedic Association proposed locomotion training (LT), an exercise program that consists primarily of one-leg standing and squatting exercises. As LT requires no specialized equipment and can be performed at a relatively low intensity, it is considered suitable for continuous practice by older adults. Another advantage of LT is the availability of standardized instructional materials, including exercise protocols and safety guidance, through the Locomo Online website, which is freely accessible to the public. These resources facilitate standardized implementation and reproducibility, making LT feasible, even in settings with limited healthcare resources and home-based environments.

LT improved various physical and health-related outcomes, including balance, quality of life (QOL), bone mineral density, and pain [6,7,8,9]. As LT is specifically designed to improve musculoskeletal function, it is expected to have a direct beneficial effect on LS. In our previous study, LT was provided to 14 community-dwelling older women and improvements in LS stage were observed in some participants [9]. Although the prior and present studies used similar LT protocols and assessed LS as an outcome, the intervention periods differed, and there was no overlap between the datasets. The prior study did not assess J-CHS, grip strength, or walking speed, and its small sample size precluded a thorough examination of the baseline characteristics of participants with favorable outcomes in LS or frailty. Regarding the potential effects of LT on frailty, LS is strongly associated with frailty and represents an important aspect of physical frailty [10,11]. Therefore, LT-associated improvements in musculoskeletal function may contribute to improvements in physical frailty, which is associated with psychological and social frailty [12]. If improved physical function enables older adults to participate more easily in daily and social activities, changes in psychological and social domains may occur. Indeed, exercise interventions for older adults reduced depressive symptoms [13] and expanded life-space mobility [14]. Based on these findings, we hypothesized that favorable changes following LT might extend beyond musculoskeletal function to include physical frailty and, potentially, the psychological and social dimensions of frailty.

Therefore, this study evaluated changes in LS and frailty following LT among community-dwelling older adults. We also explored the baseline characteristics of participants with favorable LS and frailty outcomes following the intervention.

## 2. Materials and Methods

### 2.1. Study Design

A single-arm pre–post intervention design was used to assess changes in LS and frailty following LT. The study was conducted between 18 July and 12 December 2025, with a 126-day intervention period for each participant. To minimize assessment bias, the assessors who conducted the post intervention evaluations were blinded to the baseline assessment results.

### 2.2. Ethical Considerations

The study protocol was approved by the Ethics Committee of the International University of Health and Welfare (Approval No. 24-TC-008) and registered with the University Hospital Medical Information Network Clinical Trials Registry (trial ID: UMIN000058520). The study was conducted in accordance with the Declaration of Helsinki. Before enrollment, all participants received written and verbal explanations of the study objectives and procedures, and written informed consent was obtained from each participant.

### 2.3. Participants

**Figure 1:**
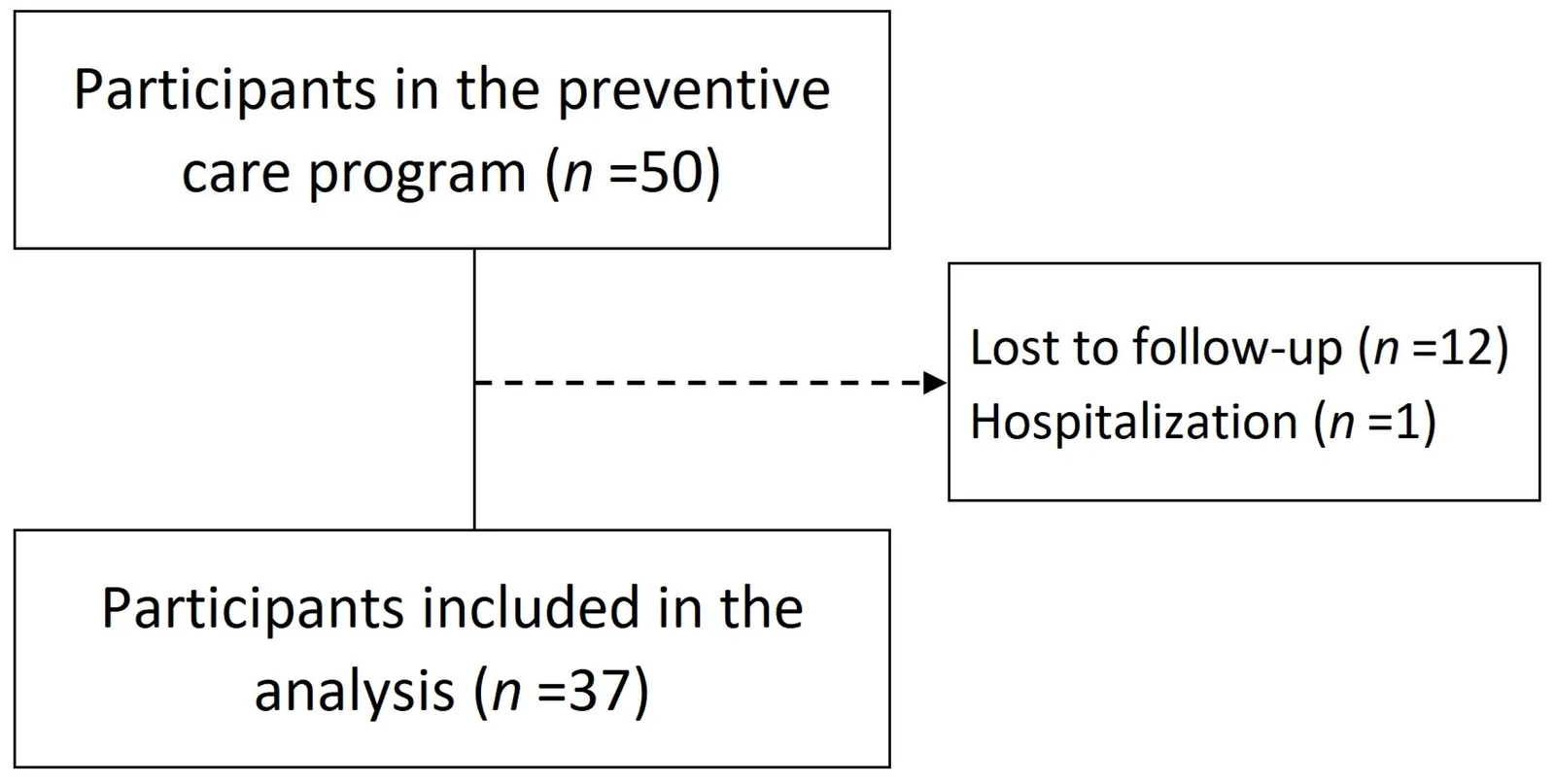
Flowchart of participant selection and enrolment.

A total of 50 community-dwelling older adults participated in a community-based preventive care program conducted in Yaita City, Tochigi Prefecture, Japan, between July and December 2025. Participants were recruited through Yaita City’s official website on a first-come, first-served basis and voluntarily enrolled in the program after being informed that the program involved exercise. No specific eligibility criteria were established regarding age, medical condition, or cognitive function. The 50 participants who were initially enrolled could perform the LT program at baseline, and none had a medical condition that prevented participation. Although the follow-up assessment was also offered on a first-come, first-served basis, 12 participants did not apply and one was hospitalized during the intervention period. Consequently, data from 37 participants (mean age, 77.6 ± 6.0 years) were included in the main analysis (Figure 1). All 37 participants included in the main analysis underwent LS and frailty assessments at baseline and follow-up. Because this study was conducted as part of a community-based preventive care program organized by Yaita City, the sample size was determined by the number of participants enrolled in the program; therefore, an a priori sample size calculation was not performed.

### 2.4. Locomotion Training

The LT program consisted of three exercises: one-leg standing, squats, and heel-raises. These exercises have been recommended as standard components of exercise programs for the prevention of locomotive syndrome [15]. At the beginning of the intervention, the participants received face-to-face instructions from a licensed physical therapist regarding the correct performance of each exercise. Subsequently, they were instructed to perform exercises daily at home. Furthermore, written instructional materials describing the exercise procedures and prescribed frequencies were provided.

#### 2.4.1. One-Leg Standing

One-leg standing exercises were performed according to the protocol described in our previous study [9]. The participants performed one set consisting of one minute of standing on each leg, three times per day [15].

#### 2.4.2. Squats

The squat exercise was performed according to the protocol described in our previous study [9]. Participants performed one set consisting of five to six repetitions three times per day [15].

#### 2.4.3. Heel Raises

The participants performed heel raises in a standing position by lifting both heels off the floor and then slowly lowering them back to the starting position. For safety, the participants were allowed to use a wall or handrail for support as needed. The exercise consisted of 10 repetitions per set, with three sets performed daily [15]. Heel raises are effective in strengthening the triceps surae muscles and have been shown to improve balance and physical function in older adults [16].

#### 2.4.4. Recording of LT Adherence

To monitor adherence to the LT program, the participants were provided with a calendar-based recording sheet during the instructional session before the intervention. The participants recorded their daily LT performance over the 126-day intervention period according to the following criteria:

O (Performed as prescribed): Completed all three exercises at the prescribed number of repetitions.

One-leg standing: 1 min on each leg × 3 sets/day.

Squats: 5–6 repetitions × 3 sets/day.

Heel raises: 10 repetitions × 3 sets/day.

△ (Partially performed): Performed fewer than the prescribed repetitions and/or sets.

✕ (Not performed): None of the three prescribed exercises were performed.

In addition, participants’ adherence to the LT program and any adverse events were verbally confirmed approximately once a month throughout the intervention period.

### 2.5. Outcome Measures

#### 2.5.1. Primary Outcome: Locomotive Syndrome

LS was assessed using the Locomotive Syndrome Risk Test, which consists of three components: the stand-up test, the two-step test, and the 25-question geriatric locomotive function scale (GLFS-25). The LS stage was determined based on the results of each component; the highest stage among the three assessments was used as the final classification.

The stand-up test was performed using platforms at heights of 10, 20, 30, and 40 cm. The participants were asked to stand up from each platform using either one or both legs. Performance was scored on a scale of 0–8, according to a previous study, with higher scores indicating better stand-up ability [17]. LS stage was classified as follows: non-LS, scores of 5–8; LS stage 1, scores of 3–4; LS stage 2, a score of 2; and LS stage 3, scores of 0–1 [15]. In the two-step test, the maximum length of two consecutive steps was measured and divided by the participant’s height to calculate the two-step test value. LS stage was classified as follows: non-LS, ≥1.3; LS stage 1, 1.1 to <1.3; LS stage 2, 0.9 to <1.1; and LS stage 3, <0.9 [15,18]. The GLFS-25 is a self-administered questionnaire consisting of 25 items assessing the physical, psychological, and social aspects of locomotive function. Higher scores indicate greater impairment of locomotive function. LS stage was classified according to previous studies as follows: non-LS, <7; LS stage 1, 7–15; LS stage 2, 16–23; and LS stage 3, ≥24 [15,18,19].

#### 2.5.2. Secondary Outcome: Frailty

Frailty was assessed using the Japanese version of the Cardiovascular Health Study (J-CHS) criteria and the Questionnaire for Medical Checkup of Old-Old (QMCOO). The J-CHS criteria assess physical frailty based on five components: weight loss (≥2 kg over the previous 6 months), self-reported exhaustion, low physical activity (no exercise at least once per week), slow usual gait speed (<1.0 m/s), and low grip strength (<28 kg for men and <18 kg for women). Participants were classified as robust (0 components), pre-frailty (1–2 components), or frailty (≥3 components) according to previous studies [20].

QMCOO is a comprehensive questionnaire designed to assess the overall health status of older adults. It comprises 15 items, each scored 0 or 1 point, yielding a total score [21,22]. Based on previous studies, participants were classified as robust (0–2 points), pre-frailty (3 points), or frailty (≥4 points) [23].

### 2.6. Group Classification According to Favorable Outcomes in LS and Frailty

To explore the characteristics of participants with favorable outcomes following the intervention, participants were classified according to changes in LS and frailty status based on previous studies [24,25,26]. For frailty, the same classification was applied using both the J-CHS and QMCOO.

Non-LS maintenance/improvement group: Participants who were classified as non-LS at both baseline and follow-up, those who had LS (LS stages 1–3) at baseline and demonstrated an improvement in at least one LS stage, or achieved non-LS status at follow-up.

Non-improvement group: Participants who did not meet the criteria for the non-LS maintenance or improvement group.

Robust maintenance/improvement group: Participants who were classified as robust at both baseline and follow-up or those who were classified as pre-frailty or frailty at baseline and demonstrated an improvement in at least one frailty category at follow-up.

Non-improvement group: Participants who did not meet the criteria for robust maintenance or improvement.

Furthermore, to distinguish improvement from maintenance of a favorable status, we conducted an additional analysis restricted to participants with LS at baseline for the LS outcome and to those with pre-frailty or frailty at baseline for the frailty outcomes. We then compared baseline characteristics between the improvement and non-improvement groups; the results are presented in the Supplementary Materials.

### 2.7. Statistical Analysis

Changes in LS and frailty outcomes before and after LT intervention were analyzed using the paired *t*-test, Wilcoxon signed-rank test, and McNemar’s test, as appropriate. Exact *p*-values were reported for all Wilcoxon signed-rank tests. Participants without pre–post change were excluded from the ranking; tied absolute differences were assigned average ranks. Effect sizes for pre–post comparisons were calculated using Cohen’s d*_z_* for paired *t*-tests, r (|Z|/√N) for Wilcoxon signed-rank tests, and φ for McNemar’s tests. Effect sizes were reported as absolute values.

As exploratory analyses to examine the characteristics of participants with favorable LS and frailty outcomes, participants were classified into a non-LS maintenance/improvement group and a non-improvement group, as well as a robust maintenance/improvement group and a non-improvement group. Baseline characteristics were compared between the groups using the independent *t*-test, Mann–Whitney *U* test, Fisher’s exact test, or chi-square test, as appropriate. In these comparisons, baseline frailty variables were examined for favorable LS outcomes, and baseline LS-related variables were examined for favorable frailty outcomes. Effect sizes for between-group comparisons were calculated using Hedges’ g for independent *t*-tests, r (|Z|/√N) for Mann–Whitney U tests, and Cramér’s V for Fisher’s exact tests and chi-square tests. Effect sizes were reported as absolute values.

In addition, a supplementary analysis was performed to compare the participants who completed the LT intervention with those who dropped out. All statistical analyses were performed using SPSS Statistics version 29 (IBM Corp., Armonk, NY, USA), and statistical significance was set at *p* < 0.05. No adjustment of *p*-values for multiple comparisons was performed, and the results of comparisons across multiple outcomes were interpreted as exploratory.

## 3. Results

Table 1 presents the changes in LS and frailty outcomes before and after LT intervention. Over the 126-day intervention period, the combined adherence rate for Performed as prescribed and Partially performed among participants who completed the intervention was 84.8 ± 17.2%. Following the 126-day LT intervention, significant improvements were observed in LS stage (*p* = 0.016), J-CHS total score (*p* = 0.014), and usual gait speed (*p* < 0.001). The mean pre–post changes (95% confidence intervals [CIs]) were +0.11 m/s (+0.05 to +0.16) for usual gait speed, −0.6 kg (−1.3 to +0.1) for grip strength, and +0.02 (−0.01 to +0.05) for the two-step test value.

**Figure 2:**
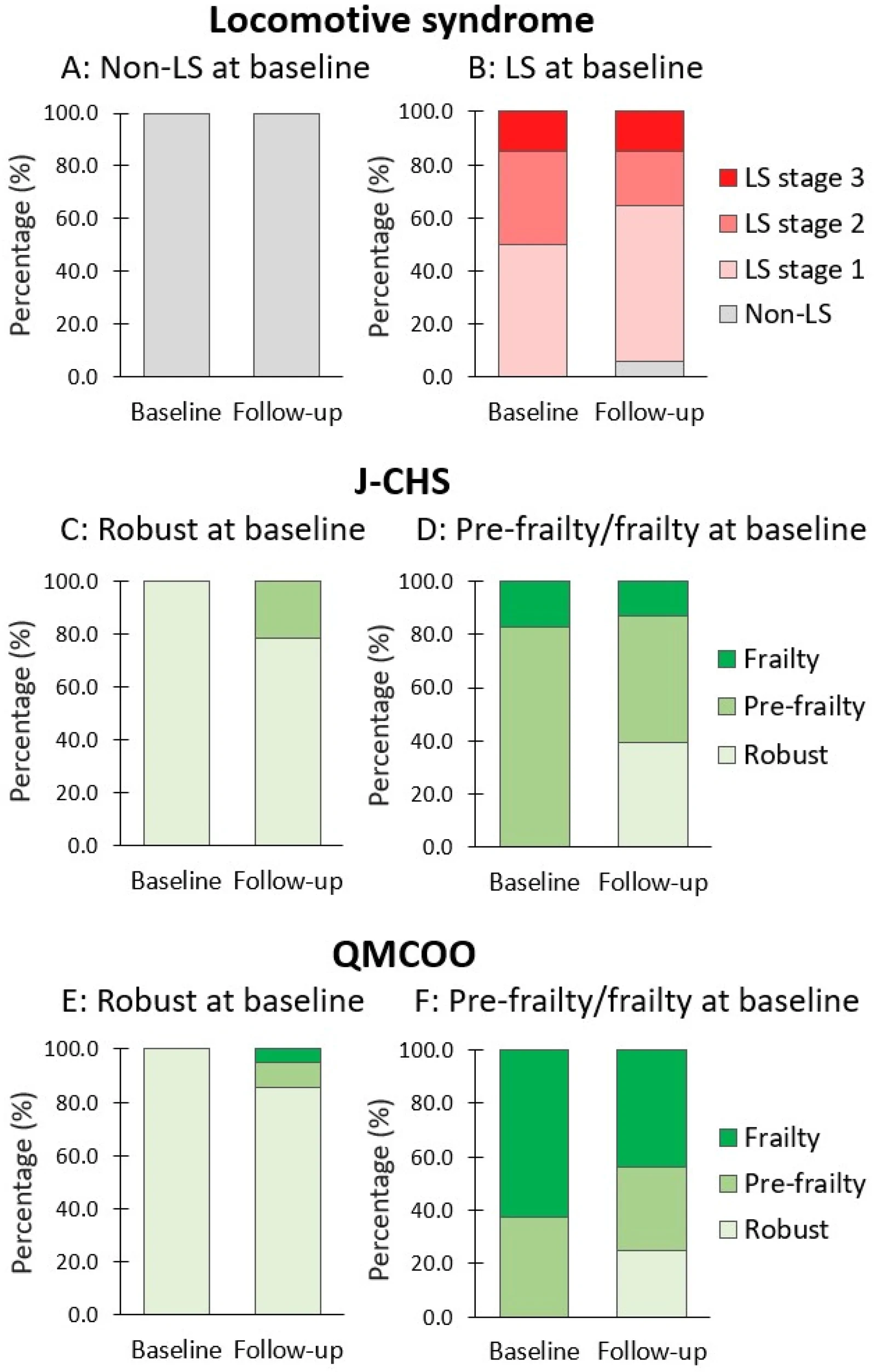
Transitions in locomotive syndrome and frailty status from baseline to follow-up after the locomotion training intervention. At the individual level, following the 126-day LT intervention, seven (20.6%) of the 34 participants with locomotive syndrome at baseline showed improvement in the locomotive syndrome stage. According to the J-CHS criteria, 10 (43.5%) of the 23 participants classified as pre-frailty or frailty at baseline showed an improvement in frailty status. According to the QMCOO, 5 (31.3%) of the 16 participants classified as pre-frailty or frailty at baseline showed an improvement in frailty status. J-CHS, Japanese version of the Cardiovascular Health Study; LS, locomotive syndrome; QMCOO, Questionnaire for Medical Checkup of Old-Old.

Figure 2 illustrates individual-level transitions in LS and frailty status before and after the intervention. Participants who were non-LS or robust at baseline are shown separately from those with LS or pre-frailty/frailty at baseline. All three participants classified as non-LS at baseline maintained their non-LS status at follow-up. Of the 34 participants with LS at baseline, seven (20.6%, 95% CI: 10.3–36.8%) showed an improvement in the LS stage, including five who improved from LS stage 2 to stage 1 and two who improved from LS stage 1 to non-LS. The remaining 27 participants maintained their baseline LS stages. According to the J-CHS, 11 of the 14 participants who were classified as robust at baseline remained robust at follow-up. Of the 23 participants classified as pre-frailty or frailty at baseline, 10 (43.5%, 95% CI: 25.6–63.2%) showed an improvement in frailty status. Specifically, nine participants improved from pre-frailty to robust status, and one improved from frailty to pre-frailty. According to the QMCOO, 18 of the 21 participants who were classified as robust at baseline remained robust at follow-up. Of the 16 participants classified as having pre-frailty or frailty at baseline, five (31.3%, 95% CI: 14.2–55.6%) experienced improvement in frailty status. Specifically, one participant improved from frailty to pre-frailty, two improved from frailty to robust status, and two improved from pre-frailty to robust status.

Table 2 presents a comparison between the non-LS maintenance/improvement and non-improvement groups. The non-LS maintenance/improvement group comprised 10 participants, whereas the non-improvement group comprised 27 participants. No significant differences were observed in baseline frailty measures or demographic characteristics between the two groups. However, the median percentage of days on which LT was performed as prescribed was higher in the non-LS maintenance/improvement group than in the non-improvement group (*p* = 0.001).

Table 3 presents a comparison between the robust maintenance/improvement and non-improvement groups based on the J-CHS. The robust maintenance/improvement group comprised 21 participants, whereas the non-improvement group comprised 16. Participants in the robust maintenance/improvement group were significantly younger (*p* = 0.027) and had significantly better two-step test values (*p* = 0.041) and GLFS-25 scores (*p* = 0.006) than those in the non-improvement group.

Table 4 presents a comparison between the robust maintenance/improvement and non-improvement groups based on QMCOO. Participants in the robust maintenance/improvement group were significantly younger (*p* < 0.001), had fewer medications (*p* = 0.024), and had significantly better LS stage (*p* = 0.010), two-step test values (*p* = 0.006), and GLFS-25 scores (*p* < 0.001) than those in the non-improvement group. Furthermore, the median percentage of days on which LT was performed as prescribed or partially was higher in the robust maintenance/improvement group than in the non-improvement group (*p* = 0.009). Additional analyses restricted to participants with LS at baseline or to those with pre-frailty or frailty at baseline showed generally similar trends (Supplementary Tables S1–S3).

Table 5 compares the baseline demographic characteristics, LS, and frailty measures between the participants who completed the LT intervention and those who dropped out of the study. No significant differences were observed between the two groups in any of the variables examined. Among the 37 participants who completed the follow-up assessment, no adverse events, including falls or worsening pain, were reported during the verbal check-ins that were conducted approximately once a month. One participant was hospitalized for a condition unrelated to LT.

## 4. Discussion

We investigated changes in LS and frailty following LT, extending the primary focus of previous studies on LS-related outcomes. At the individual level, following the intervention, 20.6% of the participants with LS at baseline showed improvement in LS stage. Improvements in frailty status were observed in 43.5% of older individuals classified as pre-frailty or frailty according to the J-CHS and in 31.3% of those classified as pre-frailty or frailty according to the QMCOO. Notably, consistent findings were obtained using both the J-CHS, which assesses physical frailty, and QMCOO, which provides a more comprehensive assessment of frailty. These findings indicate that favorable outcomes were observed for both LS and frailty following LT, despite differences in the frailty constructs that were assessed by the two instruments.

Following the 126-day LT intervention, significant improvements were observed in LS stage and usual gait speed. Although the individual components of the LS Risk Test did not show statistically significant changes, the observed improvement in LS stage may partly reflect changes across categorical thresholds. Usual gait speed improved by 0.1 m/s, which is comparable to the minimal clinically important difference generally reported for gait speed in older adults [27]. This improvement in walking speed may be related to the improvement in LS observed in the present study. Regarding individual-level frailty transitions, most participants who showed improvement according to the J-CHS improved from pre-frailty to robust, whereas only a small number of individuals improved from frailty to pre-frailty. As the J-CHS primarily reflects physical frailty, including physical performance, these findings support previous studies that highlight the importance of exercise interventions during the pre-frailty stage [28,29,30]. By contrast, according to the QMCOO, improvements were observed among participants classified as frailty or pre-frailty at baseline. Unlike the J-CHS, the QMCOO assesses multiple frailty domains, including nutritional status, cognitive function, and social participation. This broader assessment may explain the different patterns of improvement observed between the two frailty assessment tools. In addition, because the QMCOO relies solely on self-reported responses, variability in responses may contribute to changes in frailty classification.

Exploratory analyses of participants with favorable LS and frailty outcomes revealed different patterns between the two conditions. No significant differences in baseline frailty measures were observed between the non-LS maintenance/improvement and non-improvement groups. In contrast, participants in the robust maintenance/improvement group, as defined by both J-CHS and QMCOO, exhibited significantly better baseline LS measures than those in the non-improvement group. In summary, baseline frailty measures did not differ according to LS outcomes, whereas participants with favorable frailty outcomes had better baseline LS measures. However, the maintenance/improvement groups included participants who were free of LS or robust at baseline, respectively. Therefore, between-group differences in baseline characteristics may partly reflect differences in health status before the intervention. To address this, we conducted additional analyses restricted to participants with LS at baseline for the LS outcome and to those with pre-frailty or frailty at baseline for the frailty outcomes. Broadly similar patterns were observed in these analyses. The Declaration of the Medical Society for Overcoming Frailty and Locomotive Syndrome proposed that LS precedes frailty, and that the progression of LS subsequently leads to physical frailty. However, intervention studies supporting this concept are limited [4]. The present findings are consistent with this proposed relationship, although they do not establish a temporal or causal relationship between LS and frailty. Furthermore, a feature of this study is that similar trends were observed using both the J-CHS and QMCOO despite their different conceptual frameworks for assessing frailty. Moreover, these exploratory group comparisons revealed differences in age. Participants in the frailty maintenance/improvement groups defined by the J-CHS and QMCOO were younger than those in the non-improvement groups. In contrast, no significant age difference was observed between the groups defined by LS outcomes. Age is a potential confounder of the association between baseline LS status and favorable frailty outcomes. As the present findings are based on exploratory group comparisons, further research is needed to determine whether age is independently associated with these outcomes. Participants in the LS and QMCOO maintenance/improvement groups showed better adherence to LT; however, whether this association is independent of other factors requires further investigation.

In this study, no significant improvement was observed in the stand-up test. The stand-up test is known to primarily reflect lower-extremity muscle strength, particularly knee extensor strength [31]. Whereas the two-step test mainly reflects horizontal mobility, the stand-up test assesses vertical mobility [17]. Because vertical mobility has been reported to decline relatively early with age [32], a longer intervention period may be required to achieve measurable improvements. In addition, the stand-up test consists of a limited number of ordinal categories, which may have reduced its sensitivity in detecting changes following the intervention [17]. The LT program used in this study comprised body-weight exercises. Although gait performance improved following the intervention, the training load may not have been sufficient to produce substantial gains in lower-extremity muscle strength. High-intensity resistance training has been shown to be effective in improving muscle strength in older individuals [33]. Therefore, LT alone may have provided an insufficient training stimulus for relatively active older adults who voluntarily participated in this community-based preventive care program, and improvements in muscle strength depend not only on exercise but also on adequate nutritional management, particularly sufficient protein intake [34]. Taken together, future studies should examine whether combining LT with higher-intensity resistance training and nutritional interventions results in greater improvements in muscle strength and physical function among relatively active older adults such as those included in the present study.

This study had some limitations. First, this was a single-arm pre–post study without a control group, precluding causal inference regarding the observed changes following LT. Therefore, the potential influences of the Hawthorne effect, test–retest learning effects, natural functional fluctuations, and regression to the mean cannot be ruled out. Second, the participants voluntarily enrolled in a community-based preventive care program and, therefore, may have been more health-conscious than the general older adult population. In addition, the participants were recruited from a single municipality, and most were women (91.9%). These characteristics may limit the generalizability of our findings to broader community-dwelling older populations, particularly older men. In addition, because we did not collect detailed information on participants’ dietary habits, specific physical and social activities, or medical histories, we could not assess the influence of these factors on the study findings. Third, 13 of the 50 participants (26.0%) did not complete the follow-up assessment. Although comparable attrition rates have been reported in previous long-term home-based exercise interventions among community-dwelling older adults [35,36] and no significant differences in baseline characteristics were observed between participants who completed the intervention and those who dropped out (Table 5), the possibility of attrition bias cannot be excluded. The LT adherence and post-intervention status of the 12 participants who did not undergo the follow-up assessment are unknown. Fourth, although adherence to LT was relatively high in this study, adherence was assessed using self-reported records and may not fully reflect actual exercise participation. Fifth, the sample size was relatively small, particularly for subgroup comparisons, resulting in limited statistical power and precluding reliable multivariable analyses. Although the effect sizes provided additional information on the magnitude of the observed between-group differences, the findings of the subgroup analyses should be interpreted as exploratory. Despite these limitations, this study had several strengths. The participants underwent a relatively long-term LT intervention lasting 126 days, which allowed us to evaluate the changes in both LS and frailty. In addition, we explored the characteristics of participants with favorable LS and frailty outcomes following LT. Randomized controlled trials are required to verify the present findings and examine whether combining LT with higher-intensity exercise or nutritional interventions leads to further improvements in LS and frailty outcomes. Moreover, these studies should ascertain information on dietary habits, usual physical and social activities, and medical histories, and account for these factors when assessing whether the findings can be replicated across different regions and populations.

## 5. Conclusions

Following the 126-day LT program, 20.6% of participants with LS at baseline showed an improvement of at least one stage in LS. Among participants with pre-frailty or frailty at baseline, individual-level transitions to a more favorable frailty category were observed in 43.5% according to the J-CHS and 31.3% according to the QMCOO. Participants with favorable frailty outcomes had better baseline LS measures, whereas baseline frailty measures did not differ according to LS outcomes. This exploratory association is consistent with the proposed relationship that LS precedes frailty. These findings require confirmation in larger controlled studies.

## Figures and Tables

**Table 1:** Comparison of locomotive syndrome and frailty outcomes before and after locomotion training intervention.

	Baseline	Follow-Up	*p* Value	Effect Size
Locomotive syndrome				
LS stage (Non-LS/Stage 1/2/3)	3/17/12/5	5/20/7/5	0.016 * ^a^	0.44
	1.0 [1.0, 2.0] ^†^	1.0 [1.0, 2.0] ^†^		
Stand-up test, point	4.0 [3.0, 4.0]	4.0 [3.0, 4.0]	1.000 ^a^	–
Two-step test value, cm/height	1.24 (0.19)	1.26 (0.21)	0.106 ^b^	0.27
GLFS-25, point	8.0 [3.0, 15.5]	7.0 [4.0, 12.0]	0.143 ^a^	0.24
Frailty				
J-CHS				
Frailty prevalence	4 [10.8%]	3 [8.1%]	1.000 ^c^	0.16
Total score	1.0 [0, 2.0]	0.0 [0, 1.0]	0.014 * ^a^	0.42
Components				
Shrinking	4 [10.8%]	2 [5.4%]	0.687 ^c^	0.13
Weakness	5 [13.5%]	4 [10.8%]	1.000 ^c^	0.09
Exhaustion	7 [18.9%]	6 [16.2%]	1.000 ^c^	0.07
Slowness	15 [40.5%]	12 [32.4%]	0.375 ^c^	0.22
Low activity	7 [18.9%]	2 [5.4%]	0.063 ^c^	0.37
Handgrip strength, kg	24.2 (4.0)	23.6 (4.2)	0.071 ^b^	0.31
Usual walking speed, m/s	1.06 (0.22)	1.16 (0.27)	<0.001 * ^b^	0.64
QMCOO				
Frailty prevalence	10 [27.0%]	8 [21.6%]	0.625 ^c^	0.16
Total score	2.0 [1.5, 4.0]	2.0 [1.0, 3.0]	0.143 ^a^	0.24

Data presented are the mean (standard deviation), median [first and third quartiles], or count [%], as appropriate. Effect sizes were calculated using Cohen’s d*_z_* for paired *t*-tests, r (|Z|/√N) for Wilcoxon signed-rank tests, and φ for McNemar’s tests. Effect sizes are presented as absolute values. * *p* < 0.05. ^†^ LS stage was coded as follows: non-LS (0), stage 1 (1), stage 2 (2), and stage 3 (3). ^a^, Wilcoxon signed-rank test; ^b^, Paired *t*-test; ^c^, Mc Nemar’s test. GLFS, Geriatric Locomotive Function Scale; J-CHS, Japanese version of the Cardiovascular Health Study; LS, locomotive syndrome; QMCOO, Questionnaire for Medical Checkup of Old-Old.

**Table 2:** Comparison of baseline frailty between non-LS maintenance/improvement and non-improvement groups.

	Non-LS Maintenance/Improvement Group (n = 10)	Non-Improvement Group (n = 27)	*p* Value	Effect Size
Male/Female	1/9	2/25	1.000 ^a^	0.04
Age, years	76.1 (6.4)	78.1 (5.9)	0.365 ^b^	0.33
Height, cm	150.9 (4.6)	153.4 (6.4)	0.265 ^b^	0.41
Medications	2.0 [0.8, 3.0]	3.0 [1.0, 3.0]	0.489 ^c^	0.12
Frailty				
J-CHS				
Frailty	1 [10.0%]	3 [11.1%]	0.753 ^a^	0.15
Pre-frailty	4 [40.0%]	15 [55.6%]		
Robust	5 [50.0%]	9 [33.3%]		
Total score	0.5 [0, 1.3]	1.0 [0, 2.0]	0.428 ^c^	0.14
Handgrip strength, kg	25.0 (2.5)	23.9 (4.4)	0.443 ^b^	0.28
Usual walking speed, m/s	1.08 (0.22)	1.05 (0.22)	0.713 ^b^	0.13
QMCOO				
Frailty	3 [30.0%]	7 [25.9%]	1.000 ^a^	0.10
Pre-frailty	1 [10.0%]	5 [18.5%]		
Robust	6 [60.0%]	15 [55.6%]		
Total score	2.0 [1.8, 5.3]	2.0 [1.0, 4.0]	0.724 ^c^	0.06
LT adherence rate				
Performed as prescribed, %	78.2 [64.1, 87.3]	33.3 [16.7, 63.5]	0.001 * ^c^	0.50
Performed as prescribed or partially, %	100.0 [88.3, 100.0]	86.5 [65.1, 95.2]	0.010 * ^c^	0.42

Data presented are mean (standard deviation), median [first and third quartiles], or number [%], as appropriate. Effect sizes were calculated using Hedges’ g for independent *t*-tests, r (|Z|/√N) for Mann–Whitney *U* tests, and Cramér’s V for Fisher’s exact and chi-square tests. Effect sizes are presented as absolute values. * *p* < 0.05. ^a^, Fisher’s exact test; ^b^, independent *t*-test; ^c^, Mann–Whitney *U* test. J-CHS, Japanese version of the Cardiovascular Health Study; LS, locomotive syndrome; LT, locomotion training; QMCOO, Questionnaire for Medical Checkup of Old-Old.

**Table 3:** Comparison of baseline locomotive syndrome between the robust maintenance/improvement and non-improvement groups based on the J-CHS criteria.

	Robust Maintenance/Improvement Group (n = 21)	Non-Improvement Group (n = 16)	*p* Value	Effect Size
Male/Female	3/18	0/16	0.243 ^a^	0.26
Age, years	75.7 (5.2)	80.1 (6.2)	0.027 * ^b^	0.75
Height, cm	153.1 (7.0)	152.1 (4.5)	0.594 ^b^	0.17
Medications	2.0 [0.5, 3.0]	3.0 [1.3, 3.8]	0.370 ^c^	0.15
Locomotive syndrome				
LS stage (Non-LS/Stage 1/2/3)	3/10/7/1	0/7/5/4	0.108 ^c^	0.28
	1.0 [1.0, 2.0] ^†^	2.0 [1.0, 2.8] ^†^		
Stand-up test, point	4.0 [3.0, 4.0]	4.0 [3.0, 4.0]	0.916 ^c^	0.02
Two-step test value, cm/height	1.30 (0.17)	1.17 (0.20)	0.041 * ^b^	0.69
GLFS-25, point	5.0 [2.0, 11.5]	12.0 [7.3, 21.0]	0.006 * ^c^	0.44
LT adherence rate				
Performed as prescribed, %	63.5 [32.9, 81.8]	29.8 [18.5, 64.1]	0.055 ^c^	0.32
Performed as prescribed or partially, %	93.7 [84.9, 100.0]	82.2 [59.7, 95.2]	0.089 ^c^	0.29

Data presented are mean (standard deviation), median [first quartile, third quartile], or number, as appropriate. Effect sizes were calculated using Hedges’ g for independent *t*-tests, r (|Z|/√N) for Mann–Whitney *U* tests, and Cramér’s V for Fisher’s exact and chi-square tests. Effect sizes are presented as absolute values. * *p* < 0.05. ^†^ LS stage was coded as follows: non-LS (0), stage 1 (1), stage 2 (2), and stage 3 (3). ^a^, Fisher’s exact test; ^b^, independent *t*-test; ^c^, Mann–Whitney *U* test. GLFS, Geriatric Locomotive Function Scale; J-CHS, Japanese version of the Cardiovascular Health Study criteria; LS, locomotive syndrome; LT, locomotion training.

**Table 4:** Comparison of baseline locomotive syndrome between the robust maintenance/improvement and non-improvement groups based on QMCOO criteria.

	Robust Maintenance/Improvement Group (n = 23)	Non-Improvement Group (n = 14)	*p* Value	Effect Size
Male/Female	1/22	2/12	0.544 ^a^	0.18
Age, years	75.1 (5.0)	81.7 (5.4)	<0.001 * ^b^	1.27
Height, cm	151.8 (6.4)	154.1 (5.1)	0.262 ^b^	0.38
Medications	2.0 [0, 3.0]	3.0 [2.0, 4.0]	0.024 * ^c^	0.38
Locomotive syndrome				
LS stage (Non-LS/Stage 1/2/3)	3/13/6/1	0/4/6/4	0.010 * ^c^	0.45
	1.0 [1.0, 2.0] ^†^	2.0 [1.0, 3.0] ^†^		
Stand-up test, point	4.0 [3.0, 5.0]	4.0 [3.0, 4.0]	0.567 ^c^	0.10
Two-step test value, cm/height	1.30 (0.16)	1.13 (0.20)	0.006 * ^b^	0.96
GLFS-25, point	6.0 [2.0, 10.0]	15.5 [10.5, 21.5]	<0.001 * ^c^	0.57
LT adherence rate				
Performed as prescribed, %	63.5 [33.3, 81.7]	25.4 [16.7, 64.3]	0.082 ^c^	0.29
Performed as prescribed or partially, %	93.7 [85.7, 100.0]	73.0 [55.6, 95.2]	0.009 * ^c^	0.43

Data presented are mean (standard deviation), median [first quartile, third quartile], or number, as appropriate. Effect sizes were calculated using Hedges’ g for independent *t*-tests, r (|Z|/√N) for Mann–Whitney *U* tests, and Cramér’s V for Fisher’s exact and chi-square tests. Effect sizes are presented as absolute values. * *p* < 0.05. ^†^ LS stage was coded as follows: non-LS (0), stage 1 (1), stage 2 (2), and stage 3 (3). ^a^, Fisher’s exact test; ^b^, independent *t*-test; ^c^, Mann–Whitney *U* test. GLFS, Geriatric Locomotive Function Scale; LS, locomotive syndrome; LT, locomotion training; QMCOO, Questionnaire for Medical Checkup of Old-Old.

**Table 5:** Comparison of baseline data between the locomotion training completion and dropout groups.

	Completion Group (n = 37)	Dropout Group (n = 13)	*p* Value
Male/Female	3/34	1/12	1.000 ^a^
Age, years	77.6 (6.0)	77.2 (5.6)	0.849 ^b^
Height, cm	152.7 (6.0)	152.0 (6.2)	0.716 ^b^
Medications	3.0 [1.0, 3.0]	2.0 [1.0, 3.0]	0.946 ^c^
Locomotive syndrome			
LS stage (Non-LS/Stage 1/2/3)	3/17/12/5	2/8/2/1	0.157 ^c^
	1.0 [1.0, 2.0] ^†^	1.0 [1.0, 1.5] ^†^	
Stand-up test, point	4.0 [3.0, 4.0]	4.0 [3.5, 4.5]	0.220 ^c^
Two-step test value, cm/height	1.24 (0.19)	1.30 (0.11)	0.168 ^b^
GLFS-25, point	8.0 [3.0, 15.5]	6.0 [1.0, 13.5]	0.173 ^c^
Frailty			
J-CHS			
Frailty	4 [10.8%]	2 [15.4%]	0.662 ^a^
Pre-frailty	19 [51.4%]	5 [38.5%]	
Robust	14 [37.8%]	6 [46.2%]	
Total score	1.0 [0, 2.0]	1 [0, 1.5]	0.682 ^c^
Handgrip strength, kg	24.2 (4.0)	23.3 (2.7)	0.482 ^b^
Usual walking speed, m/s	1.06 (0.22)	1.05 (0.16)	0.910 ^b^
QMCOO			
Frailty	10 [27.0%]	4 [30.8%]	0.815 ^a^
Pre-frailty	6 [16.2%]	1 [7.7%]	
Robust	21 [56.8%]	8 [61.5%]	
Total score	2.0 [1.5, 4.0]	2.0 [1.0, 5.0]	0.937 ^c^

Data presented are mean (standard deviation), median [first and third quartiles], or number [%], as appropriate. ^†^ LS stage was coded as follows: non-LS (0), stage 1 (1), stage 2 (2), and stage 3 (3). ^a^, Fisher’s exact test; ^b^, independent *t*-test; ^c^, Mann–Whitney U test. GLFS, Geriatric Locomotive Function Scale; J-CHS, Japanese version of the Cardiovascular Health Study; LS, locomotive syndrome; QMCOO, Questionnaire for Medical Checkup of Old-Old.

## Data Availability

The data that support the findings of this study are available from the corresponding author upon reasonable request. The data are not publicly available due to privacy and ethical restrictions.

## Supplementary Materials

The following supporting information can be downloaded at: https://www.mdpi.com/article/10.3390/jcm15197748/s1. Table S1: Comparison of baseline frailty between the locomotive syndrome improvement and non-improvement groups; Table S2: Comparison of baseline locomotive syndrome between frailty improvement and non-improvement groups based on the J-CHS criteria; Table S3: Comparison of baseline locomotive syndrome between frailty improvement and non-improvement groups based on the QMCOO criteria.

## Abbreviations

The following abbreviations are used in this manuscript:

GLFS-25	25-question Geriatric Locomotive Function Scale
J-CHS	Japanese version of the Cardiovascular Health Study
LS	Locomotive syndrome
LT	Locomotion training
QMCOO	Questionnaire for Medical Checkup of Old-Old
QOL	Quality of life

## References

[1] Yamada M., Arai H. (2020). Long-term care system in Japan. Ann. Geriatr. Med. Res..

[2] Satake S., Arai H. (2020). Chapter 1 Frailty: Definition, diagnosis, epidemiology. Geriatr. Gerontol. Int..

[3] Nakamura K. (2008). A “super-aged” society and the “locomotive syndrome”. J. Orthop. Sci..

[4] Japanese Medical Science Federation Declaration of the Medical Society for Overcoming Frailty and Locomotive Syndrome. https://www.jmsf.or.jp/initiatives/page_792.

[5] Deguchi N., Nishimoto J., Hamada K., Akita T., Tanaka R. (2026). Locomotive syndrome severity and physical frailty progression in community-dwelling older adults: A 1-year longitudinal study from the DETECt-L study. J. Orthop. Sci..

[6] Sonobe T., Ogawa I., Seki T., Watanabe K., Kaneko Y., Yokota T., Mawatari T., Harada S., Kadowaki Y., Takenaka Y. (2026). Locomotive syndrome digital therapeutics provided via a smartphone app: Proof-of-concept single-group trial study. JMIR Aging.

[7] Aoki K., Sakuma M., Ogisho N., Nakamura K., Chosa E., Endo N. (2015). The effects of self-directed home exercise with serial telephone contacts on physical functions and quality of life in elderly people at high risk of locomotor dysfunction. Acta Med. Okayama.

[8] Mochizuki T., Yano K., Ikari K., Okazaki K. (2024). Effects of locomotion training on bone mineral density in patients with rheumatoid arthritis. Prog. Rehabil. Med..

[9] Yoshida I., Sawaya Y., Hirose T., Hanawa Y., Hirose M., Ueda K., Ito H., Urano T. (2026). Locomotion training in a care prevention program for community-dwelling older women in Japan: An exploratory pre-post intervention study. Cureus.

[10] Yoshimura N., Muraki S., Iidaka T., Oka H., Horii C., Kawaguchi H., Akune T., Nakamura K., Tanaka S. (2019). Prevalence and co-existence of locomotive syndrome, sarcopenia, and frailty: The third survey of Research on Osteoarthritis/Osteoporosis Against Disability (ROAD) study. J. Bone Miner. Metab..

[11] Ide K., Banno T., Yamato Y., Hasegawa T., Yoshida G., Yasuda T., Arima H., Oe S., Mihara Y., Ushirozako H. (2021). Relationship between locomotive syndrome, frailty and sarcopenia: Locomotive syndrome overlapped in the majority of frailty and sarcopenia patients. Geriatr. Gerontol. Int..

[12] Ye L., van Grieken A., Alhambra-Borrás T., Zhou S., Clough G., Markaki A., Bilajac L., Raat H. (2024). Interplay of physical, psychological, and social frailty among community-dwelling older adults in five European countries: A longitudinal study. J. Urban Health.

[13] Coletta G., Noguchi K.S., Beaudoin K.D., McQuarrie A., Tang A., Griffin M., Ganann R., Phillips S.M. (2024). A live online exercise program for older adults improves depression and life-space mobility: A mixed-methods pilot randomized controlled trial. PLoS ONE.

[14] Gen A., Higuchi Y., Ueda T., Hashimoto T., Kozuki W., Murakami T., Ishigami M. (2025). Intervention for social frailty focusing on physical activity and reducing loneliness: A randomized controlled trial. Clin. Interv. Aging.

[15] Locomo Online. Japanese Orthopaedic Association Locomotive Syndrome Prevention Awareness Official Website. https://locomo-joa.jp/en.

[16] Maritz C.A., Silbernagel K.G. (2016). A prospective cohort study on the effect of a balance training program, including calf muscle strengthening, in community-dwelling older adults. J. Geriatr. Phys. Ther..

[17] Ogata T., Muranaga S., Ishibashi H., Ohe T., Izumida R., Yoshimura N., Iwaya T., Nakamura K. (2015). Development of a screening program to assess motor function in the adult population: A cross-sectional observational study. J. Orthop. Sci..

[18] Sawaya Y., Hirose T., Onuma S., Nakajima R., Fujita S., Muroi S., Sato R., Yin L., Shiba T., Kobayashi K. (2024). Prevalence and associated factors of locomotive syndrome in young Japanese adults: A cross-sectional study. BMC Musculoskelet. Disord..

[19] Seichi A., Hoshino Y., Doi T., Akai M., Tobimatsu Y., Iwaya T. (2012). Development of a screening tool for risk of locomotive syndrome in the elderly: The 25-question Geriatric Locomotive Function Scale. J. Orthop. Sci..

[20] Satake S., Arai H. (2020). The revised Japanese version of the Cardiovascular Health Study criteria (revised J-CHS criteria). Geriatr. Gerontol. Int..

[21] Satake S., Arai H. (2020). Questionnaire for medical checkup of old-old (QMCOO). Geriatr. Gerontol. Int..

[22] Yakabe M., Shibasaki K., Hosoi T., Matsumoto S., Hoshi K., Akishita M., Ogawa S. (2022). Effectiveness of the Questionnaire for Medical Checkup of Old-Old (QMCOO) to diagnose frailty in community-dwelling older adults. Geriatr. Gerontol. Int..

[23] Yakabe M., Shibasaki K., Hosoi T., Matsumoto S., Hoshi K., Akishita M., Ogawa S. (2023). Validation of the questionnaire for medical checkup of old-old (QMCOO) score cutoff to diagnose frailty. BMC Geriatr..

[24] Yuan Y., Lin S., Huang X., Li N., Zheng J., Huang F., Zhu P. (2022). The identification and prediction of frailty based on Bayesian network analysis in a community-dwelling older population. BMC Geriatr..

[25] Zanforlini B.M., Trevisan C., Bertocco A., Piovesan F., Dianin M., Mazzochin M., Alessi A., Zoccarato F., Manzato E., Sergi G. (2019). Phase angle and metabolic equivalents as predictors of frailty transitions in advanced age. Exp. Gerontol..

[26] Hirose T., Sawaya Y., Ishizaka M., Hashimoto N., Kubo A., Urano T. (2024). Prevalence and factors associated with changes in frailty among community-dwelling older adults in Japan during the COVID-19 pandemic: A prospective cohort study from 2020 to 2022. Geriatr. Gerontol. Int..

[27] Morley J.E., Abbatecola A.M., Argiles J.M., Baracos V., Bauer J., Bhasin S., Cederholm T., Coats A.J., Cummings S.R., Evans W.J. (2011). Sarcopenia with limited mobility: An international consensus. J. Am. Med. Dir. Assoc..

[28] Apóstolo J., Cooke R., Bobrowicz-Campos E., Santana S., Marcucci M., Cano A., Vollenbroek-Hutten M., Germini F., D’Avanzo B., Gwyther H. (2018). Effectiveness of interventions to prevent pre-frailty and frailty progression in older adults: A systematic review. JBI Database Syst. Rev. Implement. Rep..

[29] Gordon S.J., Baker N., Kidd M., Maeder A., Grimmer K.A. (2020). Pre-frailty factors in community-dwelling 40–75 year olds: Opportunities for successful ageing. BMC Geriatr..

[30] Hirose T., Sawaya Y., Ishizaka M., Hashimoto N., Kubo A., Urano T. (2025). Has Japan overcome COVID-19 pandemic-associated frailty by 2024? Third report. J. Nutr. Health Aging.

[31] Nakamura K., Ogata T. (2016). Locomotive syndrome: Definition and management. Clin. Rev. Bone Miner. Metab..

[32] Yamada K., Ito Y.M., Akagi M., Chosa E., Fuji T., Hirano K., Ikeda S., Ishibashi H., Ishibashi Y., Ishijima M. (2020). Reference values for the locomotive syndrome risk test quantifying mobility of 8681 adults aged 20–89 years: A cross-sectional nationwide study in Japan. J. Orthop. Sci..

[33] Fiatarone M.A., O’Neill E.F., Ryan N.D., Clements K.M., Solares G.R., Nelson M.E., Roberts S.B., Kehayias J.J., Lipsitz L.A., Evans W.J. (1994). Exercise training and nutritional supplementation for physical frailty in very elderly people. N. Engl. J. Med..

[34] Coelho-Júnior H.J., Calvani R., Picca A., Tosato M., Landi F., Marzetti E. (2022). Protein intake and frailty in older adults: A systematic review and meta-analysis of observational studies. Nutrients.

[35] Månsson L., Lundin-Olsson L., Skelton D.A., Janols R., Lindgren H., Rosendahl E., Sandlund M. (2020). Older adults’ preferences for, adherence to and experiences of two self-management falls prevention home exercise programmes: A comparison between a digital programme and a paper booklet. BMC Geriatr..

[36] van den Helder J., Mehra S., van Dronkelaar C., ter Riet G., Tieland M., Visser B., Kröse B.J.A., Engelbert R.H.H., Weijs P.J.M. (2020). Blended home-based exercise and dietary protein in community-dwelling older adults: A cluster randomized controlled trial. J. Cachexia Sarcopenia Muscle.

