# Supplementary material for: Characteristics of Older Adults with Favorable Locomotive Syndrome and Frailty Outcomes Following Locomotion Training: A Preliminary Pre–Post Study
Source: J Clin Med. 2026 Oct 8;15(19):7748. doi: 10.3390/jcm15197748 (PMC13665866; doi:10.3390/jcm15197748)
Supplement: Supplementary file 1 [file jcm-15-07748-s001.zip › jcm-4558667-supplementary.pdf]

Table S1. Comparison of baseline frailty between the locomotive syndrome improvement and non-improvement groups.

	LS improvement group (n=7)	Non-improvement group (n=27)	*p* Value	Effect size
Male/Female	1/6	2/25	0.511 [a]	0.10
Age, years	77.4 (7.2)	78.1 (5.9)	0.784 [b]	0.11
Height, cm	152.2 (4.6)	153.4 (6.4)	0.662 [b]	0.18
Medications	3.0 [2.0, 3.0]	3.0 [1.0, 3.0]	1.000 [c]	0.004
Frailty				
J-CHS				
Frailty	1 [14.3%]	3 [11.1%]		
Pre-frailty	3 [42.9%]	15 [55.6%]	0.850 [a]	0.10
Robust	3 [42.9%]	9 [33.3%]		
Total score	1.0 [0, 2.0]	1.0 [0, 2.0]	0.803 [c]	0.05
Handgrip strength, kg	25.6 (2.6)	23.9 (4.4)	0.325 [b]	0.41
Usual walking speed, m/sec	1.01 (0.21)	1.05 (0.22)	0.693 [b]	0.17
QMCOO				
Frailty	3 [42.9%]	7 [25.9%]		
Pre-frailty	1 [14.3%]	5 [18.5%]	0.846 [a]	0.15
Robust	3 [42.9%]	15 [55.6%]		
Total score	3.0 [2.0, 6.0]	2.0 [1.0, 3.0]	0.314 [c]	0.18
LT adherence rate				
Performed as prescribed, %	80.2 [63.5, 86.5]	33.3 [16.7, 63.5]	0.013* [c]	0.42
Performed as prescribed or partially, %	100.0 [84.1, 100.0]	86.5 [65.1, 95.2]	0.097 [c]	0.29

This analysis was restricted to participants with LS at baseline; three participants without LS at baseline were excluded.

Data presented are mean (standard deviation), median [first and third quartiles], or number [%], as appropriate. Effect sizes were calculated using Hedges' g for independent *t*-tests, r ($|Z|/\sqrt{N}$) for Mann–Whitney *U* tests, and Cramér's V for Fisher's exact and chi-square tests. Effect sizes are presented as absolute values.

* $p<0.05$.

a, Fisher’s exact test; b, independent *t*-test; c, Mann–Whitney *U* test.

J-CHS, Japanese version of the Cardiovascular Health Study; LS, locomotive syndrome; LT, locomotion training; QMCOO, Questionnaire for Medical Checkup of Old-Old.

Table S2. Comparison of baseline locomotive syndrome between frailty improvement and non-improvement groups based on the J-CHS criteria.

	**Improvement group (n=10)**	**Non-improvement group (n=13)**	***p* Value**	**Effect size**
Male/Female	3/7	0/13	0.068 [a]	0.44
Age, years	77.2 (6.6)	81.5 (5.8)	0.108 [b]	0.68
Height, cm	156.6 (7.8)	150.8 (3.7)	0.055 [b]	0.94
Medications	3.0 [0.8, 3.3]	3.0 [1.5, 3.5]	0.784 [c]	0.06
Locomotive syndrome				
LS stage (Non-LS/Stage 1/2/3)	1/4/4/1<br>1.5 [1.0, 2.0] †	0/5/4/4<br>2.0 [1.0, 3.0] †	0.343 [c]	0.22
Stand-up test, point	3.0 [3.0, 4.0]	4.0 [2.5, 4.5]	0.446 [c]	0.17
Two-step test value, cm/height	1.25 (0.18)	1.15 (0.21)	0.285 [b]	0.44
GLFS-25, point	8.5 [2.8, 13.8]	15.0 [9.0, 22.0]	0.077 [c]	0.38
LT adherence rate				
Performed as prescribed, %	66.7 [26.2, 81.0]	24.6 [13.1, 54.0]	0.088 [c]	0.36
Performed as prescribed or partially, %	94.9 [84.9, 100.0]	80.2 [56.8, 94.5]	0.101 [c]	0.35

This analysis was restricted to participants classified as having pre-frailty or frailty according to the J-CHS criteria at baseline; therefore, 14 participants classified as robust at baseline were excluded.

Data presented are mean (standard deviation), median [first quartile, third quartile], or number, as appropriate. Effect sizes were calculated using Hedges' g for independent *t*-tests, r (|Z|/√N) for Mann–Whitney *U* tests, and Cramér's V for Fisher's exact and chi-square tests. Effect sizes are presented as absolute values.

* $p<0.05$.

† LS stage was coded as follows: non-LS (0), stage 1 (1), stage 2 (2), and stage 3 (3).

a, Fisher's exact test; b, independent *t*-test; c, Mann–Whitney *U* test.

GLFS, Geriatric Locomotive Function Scale; J-CHS, Japanese version of the Cardiovascular Health Study criteria; LS, locomotive syndrome; LT, locomotion training.

Table S3. Comparison of baseline locomotive syndrome between frailty improvement and non-improvement groups based on the QMCOO criteria.

	**Improvement group (n=5)**	**Non-improvement group (n=11)**	***p* Value**	**Effect size**
Male/Female	0/5	2/9	1.000 [a]	0.25
Age, years	73.0 (2.9)	82.6 (5.6)	0.003* [b]	1.82
Height, cm	155.0 (7.0)	154.0 (5.7)	0.754 [b]	0.16
Medications	2.0 [1.0, 3.0]	3.0 [3.0, 4.0]	0.090 [c]	0.46
Locomotive syndrome				
LS stage (Non-LS/Stage 1/2/3)	0/4/1/0<br>1.0 [1.0, 1.5] †	0/2/5/4<br>2.0 [2.0, 3.0] †	0.027* [c]	0.57
Stand-up test, point	4.0 [3.0, 4.5]	3.0 [3.0, 4.0]	0.377 [c]	0.26
Two-step test value, cm/height	1.42 (0.06)	1.09 (0.20)	<0.001* [b]	1.84
GLFS-25, point	8.0 [6.0, 14.5]	16.0 [9.0, 23.0]	0.145 [c]	0.37
LT adherence rate				
Performed as prescribed, %	63.5 [56.8, 84.2]	23.8 [16.7, 36.5]	0.027* [c]	0.55
Performed as prescribed or partially, %	89.7 [84.9, 98.8]	65.1 [55.6, 80.2]	0.038* [c]	0.53

This analysis was restricted to participants classified as having pre-frailty or frailty according to the QMCOO criteria at baseline; therefore, 21 participants who were classified as robust at baseline were excluded.

Data presented are mean (standard deviation), median [first quartile, third quartile], or number, as appropriate. Effect sizes were calculated using Hedges' g for independent *t*-tests, r (|Z|/√N) for Mann–Whitney *U* tests, and Cramér's V for Fisher's exact and chi-square tests. Effect sizes are presented as absolute values.

* $p<0.05$.

† LS stage was coded as follows: non-LS (0), stage 1 (1), stage 2 (2), and stage 3 (3).

a, Fisher's exact test; b, independent *t*-test; c, Mann–Whitney *U* test.

GLFS, Geriatric Locomotive Function Scale; LS, locomotive syndrome; LT, locomotion training; QMCOO, Questionnaire for Medical Checkup of Old-Old.
